# Immune-Boosting and Antiviral Effects of Antioxidants in COVID-19 Pneumonia: A Therapeutic Perspective

**DOI:** 10.3390/life15010113

**Published:** 2025-01-16

**Authors:** Stefano Sanduzzi Zamparelli, Alessandro Sanduzzi Zamparelli, Marialuisa Bocchino

**Affiliations:** 1 Division of Pneumology and Semi-Intensive Respiratory Therapy, A. Cardarelli Hospital, 80131 Naples, Italy; 2 Department of Clinical Medicine and Surgery, University of Naples “Federico II”, 80131 Naples, Italy; sanduzzi@unina.it (A.S.Z.); marialuisa.bocchino@unina.it (M.B.); 3 UNESCO Chair for Health Education and Sustainable Development, University of Naples “Federico II”, 80131 Naples, Italy; 4 ERN Lung, 60596 Frankfurt am Main, Germany

**Keywords:** COVID-19 pneumonia, antioxidants, nitric oxide, melatonin, ozone, vitamin D, vitamin C, N-acetylcysteine, polyphenols

## Abstract

The COVID-19 pandemic caused by Severe Acute Respiratory Syndrome Coronavirus 2 (SARS-CoV-2) has profoundly impacted global health, with pneumonia emerging as a major complication in severe cases. The pathogenesis of COVID-19 is marked by the overproduction of reactive oxygen species (ROS) and an excessive inflammatory response, resulting in oxidative stress and significant tissue damage, particularly in the respiratory system. Antioxidants have garnered considerable attention for their potential role in managing COVID-19 pneumonia by mitigating oxidative stress and modulating immune responses. This review provides a comprehensive overview of the literature on the use of antioxidants in hospitalized patients with mild-to-moderate COVID-19. Studies exploring antioxidants, including vitamins, trace elements, nitric oxide (NO), ozone (O_3_), glutathione (GSH), L-carnitine, melatonin, bromelain, N-acetylcysteine (NAC), and numerous polyphenols, have yielded promising outcomes. Through their ROS-scavenging properties, these molecules support endothelial function, reduce the thrombosis risk, and may help mitigate the effects of the cytokine storm, a key contributor to COVID-19 morbidity and mortality. Clinical evidence suggests that antioxidant supplementation may improve patient outcomes by decreasing inflammation, supporting immune cell function, and potentially shortening recovery times. Furthermore, these molecules may mitigate the symptoms of COVID-19 by exerting direct antiviral effects that inhibit the infection process and genomic replication of SARS-CoV-2 in host cells. Moreover, antioxidants may work synergistically with standard antiviral treatments to reduce viral-induced oxidative damage. By integrating findings from the literature with real-world data from our clinical experience, we gain a more profound understanding of the role of antioxidants in managing COVID-19 pneumonia. Further research combining comprehensive literature reviews with real-world data analysis is crucial to validate the efficacy of antioxidants and establish evidence-based guidelines for their use in clinical practice.

## 1. Introduction

Severe Acute Respiratory Syndrome Coronavirus 2 (SARS-CoV-2), identified as the causative agent of Coronavirus Disease 2019 (COVID-19), was first documented in Wuhan, China, in December 2019. It rapidly evolved into a global pandemic, representing a significant public health challenge [1]. By July 2024, the virus had infected over 775.83 million people and caused more than 7.06 million deaths globally. Despite the World Health Organization (WHO) declaring the pandemic over in May 2023, SARS-CoV-2 continues to circulate, resulting in ongoing transmission and loss of lives [2]. SARS-CoV-2 infection presents a wide spectrum of clinical manifestations, ranging from asymptomatic cases to mild respiratory symptoms, and in some cases, severe or life-threatening respiratory distress [3]. While swab tests remain the primary diagnostic method for COVID-19, several biomarkers, including Krebs von den Lungen-6 (KL-6), C-reactive protein (CRP), interleukin-6 (IL-6), and SARS-CoV-2 Nucleocapsid protein (Nag), show potential for early detection, even in asymptomatic or minimally symptomatic individuals [4,5,6].

### 1.1. SARS-CoV-2 Structure and Molecular Mechanisms of COVID-19 Pathogenesis

SARS-CoV-2, similar to SARS-CoV and Middle East respiratory syndrome coronavirus (MERS-CoV), is categorized within the Betacoronavirus genus, which comprises a large family of positive-sense, enveloped, highly diverse, and single-stranded RNA viruses. The SARS-CoV-2 genome, ranging from approximately 26,000 to 32,000 bases, features a 5′-cap structure and a 3′-poly-A tail encoding 29 proteins, including 25 putative accessory and non-structural proteins (NSPs), and 4 structural proteins. NSPs are essential for viral RNA replication and immune evasion; accessory proteins facilitate viral infection and survival, while structural proteins assemble mature viral particles. The 5′-proximal two-thirds of the coronavirus genome contain the replicase gene, which encodes two open reading frames (ORF1a and ORF1b) while the 3′ end encodes various ORFs, including those for structural proteins, such as glycoprotein (S), membrane (M), envelope (E), and nucleocapsid (N) [7].

The S protein, which consists of two subunits, plays an essential role in the virus’s entry into host cells: the S1 subunit is responsible for binding to the host receptor angiotensin-converting enzyme 2 (ACE2), while the S2 subunit facilitates the fusion of viral and host membranes. The junction between S1 and S2 includes a furin cleavage site, cleaved within cells producing the virus. Following the binding to ACE2, the S protein undergoes further cleavage by the transmembrane serine protease 2 (TMPRSS2) at the S2′ site. This process activates the S2 subunit, enabling the fusion of the viral and host membranes and allowing the viral ribonucleoprotein to enter the host cell. An alternative pathway for viral entry involves endosomal cleavage by cathepsins, although this mechanism is less effective in primary epithelial cells. Additionally, other co-receptors such as neuropilin 1, dipeptidyl peptidase-4 (DPP-4), and proteases, including cathepsin L, TMPRSS11D, and TMPRSS13, may contribute to the entry of SARS-CoV-2 [8]. Following the membrane fusion of SARS-CoV-2, the viral nucleocapsid disassembles, releasing the positive-sense RNA genome into the host cell cytoplasm. This RNA is translated by host ribosomes into two polyproteins, pp1a and pp1b, which are subsequently cleaved by the papain-like protease (PLpro) and the 3C-like protease (3CLpro) to produce NSPs, including the critical RNA-dependent RNA polymerase (RdRp, NSP-12), essential for transcription and translation. RdRp, along with NSP-7 and NSP-8, forms the replicase–transcriptase complex (RTC) responsible for replicating genomic and sub-genomic RNAs. PLpro specifically cleaves pp1a to generate NSPs vital for viral functions, whereas 3CLpro cleaves both polyproteins to yield additional NSPs necessary for the viral life cycle. Newly synthesized envelope glycoproteins in the endoplasmic reticulum or Golgi apparatus aid in nucleocapsid formation with genomic RNA, leading to the release of viral particles through plasma membrane fusion. Once the virus enters cells, its antigens are processed and presented by antigen-presenting cells (APCs) through major histocompatibility complex (MHC) molecules, primarily MHC I, with MHC II also playing a role. Following infection, APCs induce both humoral and cellular immune responses via virus-specific B and T cells, leading to the generation of antibodies such as IgG and IgM against SARS-CoV-2 [9]. Viral infection generates Pathogen-Associated Molecular Patterns (PAMPs) and Damage-Associated Molecular Patterns (DAMPs) detected by Pattern Recognition Receptors (PRRs), activating downstream transcription factors like Interferon Regulatory Factor-3 (IRF-3) and Kappa Light Chains of Activated B-cell Nuclear Enhancer Factor (NF-κB). These factors lead to the expression of type I and III interferons (IFNs) and various pro-inflammatory agents, including cytokines such as Tumor Necrosis Factor α (TNF-α), Transforming Growth Factor β (TGF-β), IL-1β, IL-6, IL-8, IL-12, IL-18 and chemokines such as Chemokine Ligand-2 (CCL-2), CCL-3, CCL-5, and Chemokine Ligand Motif C-X-C-8 (CXCL-8), CXCL-9, and CXCL-10. The released pro-inflammatory substances recruit immune cells such as monocytes, natural killer (NK) cells, neutrophils, and macrophages, which further secrete cytokines and generate reactive oxygen species (ROS), creating an inflammatory feedback loop that exacerbates tissue damage [10].

**Figure 1:**
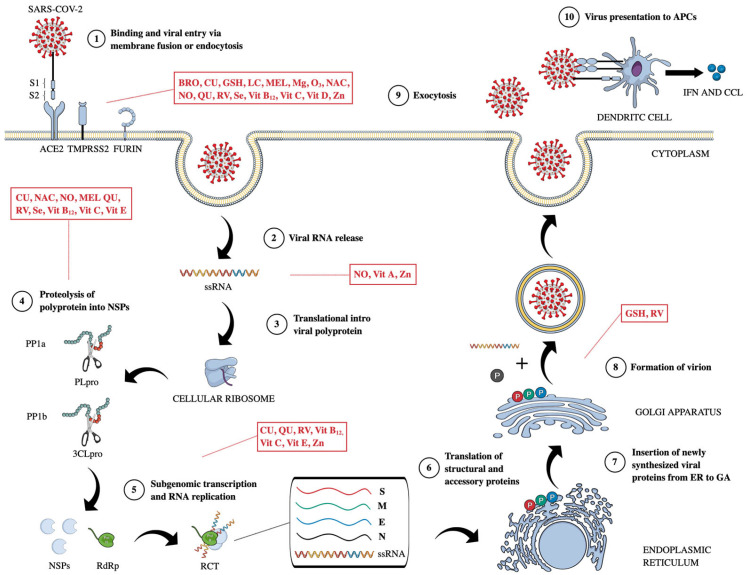
SARS-CoV-2 infection cycle and main direct antiviral effects of antioxidants. Initially, the virus binds to the host cell thanks to the interaction between spike (S) protein with the angiotensin-converting enzyme 2 (ACE2) receptor, a process facilitated by the proteolytic action of furin and transmembrane serine protease 2 (TMPRSS2). This binding is enhanced by cellular proteases such as TMPRSS2 and furin. Subsequently, the virus enters the cell through endosomal membrane fusion, releasing its RNA genome into the cytoplasm. Once inside, SARS-CoV-2 exploits the host’s ribosomes to translate its RNA into polyproteins pp1a and pp1b, which are cleaved by the proteases papain-like protease (PLpro) and 3C-like protease (3CLpro) to produce non-structural proteins (NSPs), including the essential RNA-dependent RNA polymerase (RdRp). This latter one is essential for forming the replicase–transcriptase complex (RTC) for the transcription and translation of its genomic material. Newly synthesized envelope glycoproteins facilitate nucleocapsid assembly with genomic RNA, releasing viral particles via plasma membrane fusion. This process results in the production of both structural (S, membrane [M], envelope [E], and nucleocapsid [N] proteins) and NSP viral proteins, which, along with genomic RNA, are assembled into new virions. Finally, the newly formed virions are transported via vesicles and released from the infected cell, spreading the virus to adjacent cells. The figure illustrates the various mechanisms by which the molecules examined in this review directly inhibit the entry or replication of SARS-CoV-2. For significant details of the precise antiviral mechanism of antioxidants, consult the appropriate section in the text. Abbreviations: BRO, bromelain; CU, curcumin; GSH, glutathione; LC, L-carnitine; Mg, magnesium; MEL, melatonin; O_3_, ozone; NAC, n-acetylcysteine; NO, nitric oxide; QU, quercetin; RV, resveratrol; Se, selenium; Vit, vitamin; Zn, Zinc [created with mindthegraph.com].

Many clinical manifestations of SARS-CoV-2 infection are linked to virus-induced changes in the immune system and consequent tissue damage. A dysregulated immune response leads to the overproduction of pro-inflammatory cytokines, known as a cytokine storm, which directly, indirectly, or synergistically can lead to lung injury, acute respiratory distress syndrome (ARDS), and multi-organ damage in COVID-19 patients [11] (Figure 1).

### 1.2. Clinical Course and Therapy of COVID-19

The symptoms of SARS-CoV-2 infection typically emerge around 5.2 days post-exposure, commonly including cough, fatigue, and fever, and may also present additional signs such as headache, lymphopenia, dyspnea, and gastrointestinal disturbances. In severe cases of COVID-19, complications such as pneumonia, acute cardiac injury, respiratory failure, and ARDS can occur, potentially leading to increased mortality and predisposition to thromboembolic diseases due to factors like immobilization and inflammation [12]. Although most patients present only mild symptoms, the spectrum of COVID-19 clinical course is highly heterogeneous, ranging from asymptomatic or paucisymptomatic infection to fatal ARDS. According to the WHO, COVID-19 severity classifications for SARS-CoV-2-positive patients include asymptomatic patients with no symptoms, mild cases with symptoms but no viral pneumonia or hypoxia, moderate cases showing pneumonia symptoms without respiratory distress and with oxygen saturation (SpO_2_) ≥ 90%, severe cases exhibiting severe pneumonia signs or respiratory distress with a respiratory rate over 30 breaths per minute or SpO_2_ < 90%, and critical cases requiring intensive care unit (ICU) support, including those with ARDS, multi-organ failure, or septic shock. Data from the first and second wave indicated that amongst symptomatic patients, a majority experienced either mild (40%) or moderate (40%) disease, while approximately 15% and 5% developed severe and critical disease, respectively [13]. Although the treatment for hospitalized patients with COVID-19 can vary based on the severity of their condition, systemic corticosteroids and anticoagulation are still the cornerstone in patients requiring supplementary oxygen or ventilatory support. Since the pandemic started, COVID-19 therapy has made remarkable progress, introducing monoclonal anti-IL-6 antibodies such as tocilizumab or anti-IL-1R antibodies such as Anakinra; inhibitors of Janus kinase (JAK) such as baricitinib; and different antivirals including remdesivir, molnupiravir, nirmatrelvir/ritonavir, casirivimab/imdevimab, bamlanivimab/etesevimab, and tixagevimab/cilgavimab [14]. Although the COVID-19 mortality rates have fluctuated since the early phases of the pandemic due to modifications in the virus and changes in standard care practices, they have remained high among patients hospitalized with SARS-CoV-2 with rates of 5.70% [15].

### 1.3. Role of the Immune System and Oxidative Stress

Severe COVID-19 is linked to excessive pro-inflammatory cytokine release due to dysregulated innate and adaptive immune responses [16]. This hyperactivation leads to the recruitment and activation of inflammatory cells, such as macrophages and neutrophils, and contributes to vascular endothelial dysfunction [11]. Oxidative stress, characterized by an imbalance between ROS and antioxidant defenses, plays a key role in disease progression. The increase in ROS or a reduction in antioxidant defenses exacerbates vascular damage, endothelial dysfunction, and reduced nitric oxide (NO) production, further impairing vasodilation and contributing to thrombosis [17,18]. Severe COVID-19 patients often exhibit low levels of nitrite/nitrate, by-products of NO metabolism, which may worsen vasodilatory dysfunction and promote organ failure [19]. The oxidative stress-driven inflammatory response creates a vicious cycle of neutrophil infiltration and cytokine production, leading to tissue damage, hypoxia, and ARDS. This cascade is fueled by increased ROS levels, reduced antioxidant enzyme activity, and disruption of the redox balance. Inflammatory pathways, such as the NF-κB pathway, amplify the production of cytokines such as IL-6, IL-1β, IL-10, and TNF-α, worsening ARDS [20,21]. The reduction in ROS levels is ensured by an antioxidant defense system comprising both enzymatic and non-enzymatic components [17]. Non-enzymatic antioxidants are primarily obtained through the diet and include cobalamin (vitamin B_12_); ascorbic acid (vitamin C); calciferol (vitamin D); α-tocopherol (vitamin E); carotenoids such as beta-carotene (vitamin A); flavonoids; and isoprenoids like ubiquinone and plastoquinone. Endogenous non-enzymatic antioxidants include compounds like glutathione (GSH), melatonin, bilirubin, and uric acid [22]. On the other hand, enzymatic antioxidants are synthesized within the body. Key enzymes include superoxide dismutase (SOD), which catalyzes the conversion of superoxide radicals into oxygen and hydrogen peroxide; catalase (CAT), which breaks down hydrogen peroxide (H_2_O_2_) into water and oxygen; and GSH peroxidase (GPx), which neutralizes H_2_O_2_ using GSH as a reducing agent [23].

### 1.4. Micronutrient Deficiency and Immune Function

Nutritional deficiencies can impair immune responses, reduce cytokine and antibody production, and compromise defenses against viral infections [24]. Low plasma levels of antioxidants, including vitamin A, vitamin C, vitamin D, selenium (Se), magnesium (Mg) and zinc (Zn), are associated with poor outcomes in respiratory infections, including COVID-19. Vitamin A maintains mucosal integrity, regulates inflammation, and supports T cell function. Zn, a cofactor for over 300 enzymes, modulates immune responses and reduces oxidative stress [25]. Se contributes to antioxidant enzyme activity, and its deficiency exacerbates respiratory dysfunction [26]. Deficiencies in these and other micronutrients may also increase the risk of bacterial superinfections in COVID-19, worsening respiratory damage and mortality [27]. COVID-19 has been shown to alter micronutrient levels through mechanisms like hypoxia and IL-6-mediated suppression of selenoprotein (Sel) expression, further impairing antioxidant defenses and increasing ROS generation [28].

### 1.5. Endothelial Dysfunction

The endothelium, a critical regulator of blood flow and thrombotic balance, is significantly affected in COVID-19. SARS-CoV-2 directly interacts with endothelial cells, promoting inflammation, oxidative stress, and endothelitis. These processes lead to increased permeability, impaired vasodilation, and apoptosis, contributing to vascular dysfunction. Chronic inflammation and redox imbalance exacerbate these effects, as seen in cardiovascular and cerebrovascular diseases. Even after recovery, convalescent patients often exhibit impaired endothelial function, evidenced by reduced flow-mediated dilation (FMD) and elevated markers of inflammation, such as IL-6 and endothelin-1 [29].

### 1.6. Therapeutic Potential of Antioxidants

Given the critical role of oxidative stress in COVID-19 pathogenesis, antioxidant supplementation offers a promising strategy to mitigate disease severity. Numerous antioxidants, including vitamins A, B_12_, C, D, and E, as well as NO, ozone (O_3_), GSH, L-carnitine, melatonin, bromelain, and N-acetylcysteine (NAC), have been demonstrated to exhibit antiviral, anti-inflammatory, and immune-enhancing properties. Additionally, trace elements such as Zn, Mg, and Se, or polyphenolic compounds including resveratrol, quercetin, and curcumin, contribute to these beneficial effects. Certain therapies targeting redox imbalance, including ubiquinol, mitoquinone mesylate (MitoQ: Auckland, New Zealand), and nuclear factor erythroid-related factor-2 (NRF-2) agonists, have shown potential in preclinical studies. However, these interventions require further validation through randomized controlled trials.

As SARS-CoV-2 continues to circulate globally, this review seeks to present a comprehensive overview of the primary antioxidants employed in conjunction with standard care for the treatment of hospitalized patients recovering from mild-to-moderate COVID-19, focusing on their mechanisms of action and key effects.

## 2. Modulators of Cellular Biochemistry

### 2.1. Glutathione

GSH is the most abundant antioxidant and a key detoxification agent in cells, essential for processes associated with thiol-redox status maintenance. It is synthesized in the cytoplasm through a 2 ATP-dependent reaction catalyzed by γ-glutamyl-cysteine synthetase (GCS) and GSH synthetase. GSH, a tripeptide consisting of cysteine, glycine, and glutamic acid, has a highly active thiol group (–SH) that is prone to oxidation, resulting in the oxidized form of glutathione disulfide (GSSG). The GSH redox cycle, involving the conversion of hydrogen peroxide to water by GPx and the regeneration of GSH from GSSG by glutathione reductase using nicotinamide adenine dinucleotide phosphate hydrogen (NADPH), is vital for cellular defense against oxidative stress and may help mitigate COVID-19 severity. GSH is a primary antioxidant, directly scavenging various free radicals and detoxifying harmful substances like hydrogen peroxides and lipid peroxyl radicals. GSH is essential for immune system function, particularly lymphocyte activity, as its deficiency can impair T cell proliferation and immune responses, resulting in various diseases such as viral infections, cancer, and diabetes. It plays an essential role in innate and adaptive immunity by modulating T lymphocyte proliferation, enhancing neutrophil phagocytosis, and supporting dendritic cell functions. GSH is integral to the antigen presenting process in macrophages and dendritic cells by facilitating the degradation of protein antigens essential for T cell activation, promoting interferon-gamma production. Furthermore, GSH depletion can shift the cytokine response from T helper 1 (Th1) to Th2, impairing cell-mediated immunity. Moreover, GSH scavenges ROS and enhances macrophage polarization, indicating its potential as a strategy to bolster the human immune defense system. GSH supports cytotoxic T cell activation, and its depletion is associated with lymphopenia and impaired immunity, which can lead to severe COVID-19 outcomes. Low GSH levels hinder interleukin-2 production, reducing lymphocyte proliferation [30]. Furthermore, GSH depletion can trigger lymphocyte apoptosis and ferroptosis, contributing to COVID-19-related complications. Therefore, maintaining adequate GSH levels is vital for effective immune responses and managing viral infections. SARS-CoV-2 infection induces oxidative stress and inflammation, leading to diminished GSH levels, which exacerbate severe COVID-19 outcomes and conditions like ARDS by triggering cytokine storms and disrupting the ACE/ACE2 equilibrium, ultimately resulting in respiratory failure. This shift favors ACE activity, resulting in increased vasoconstriction, oxidative stress, inflammation, and apoptosis. In contrast, GSH plays a protective role by reducing ROS production, activating the ACE2 pathway, inhibiting the activation of NF-kB, and aiding in the management of the cytokine storm [31]. Dewan et al. [32] evaluated the impact of administering a loading dose of 2400 mg of GSH, followed by intravenous injections of 1200 mg every 12 h over seven days, or until clinical improvement was observed, in patients with moderate COVID-19. Compared to the placebo group, a significant clinical improvement was observed in the GSH group (*p* < 0.001) during the initial treatment days. Patients receiving GSH also experienced a reduction in clinical severity and intensity of ventilation support, and a reduction in hospitalization.

### 2.2. L-Carnitine

Carnitine exists in two forms, D-carnitine and L-carnitine, with only L-carnitine being biologically active. L-carnitine, a trimethylated amino acid similar to choline, acts as a cofactor in the conversion of long-chain fatty acids to acylcarnitine, facilitating their transport across the inner mitochondrial membrane into the matrix for β-oxidation leading to energy production via the Krebs cycle. This transport is primarily facilitated by the carnitine palmitoyltransferase (CPT) system, which has three tissue-specific isoforms. Approximately 75% of L-carnitine is sourced from the diet, while the remainder is synthesized from lysine and methionine in the liver and kidneys, with 95% stored in skeletal muscle and smaller amounts in the brain, heart, and sperm. Lower levels of L-carnitine are observed in elderly individuals and those suffering from chronic diseases, which may enhance their susceptibility to chronic inflammation. This bioactive compound may play a significant role in inflammatory diseases by modulating cell inflammatory responses, potentially reducing key inflammatory cytokines such as TNF-α and IL-6. L-carnitine serves as an antioxidant and anti-inflammatory agent by decreasing malondialdehyde (MDA), a marker for oxidative stress, and increasing GSH and SOD levels, protecting cells from free radicals. It also modulates immune and nervous system functions while inhibiting inflammatory factor expression. Its immune-boosting effects include the enhancement of neutrophil and macrophage functions through the modulation of glucose 6-phosphate dehydrogenase and macrophage inhibitory factor-1 (MIF-1) production. Moreover, L-carnitine may reduce lymphocyte apoptosis by downregulating pro-apoptotic Fas signaling and ceramide production, potentially mitigating cytokine storms. L-carnitine supplementation has shown increased CD4+ and CD8+ T cell numbers and lowered TNF-α levels. Additionally, L-carnitine reduces leukotriene synthesis by inhibiting lipoxygenase activity, thus alleviating lung inflammation [33]. Recent studies indicate a negative correlation between carnitine levels and COVID-19 susceptibility, where higher levels correlate with reduced vulnerability. Additionally, L-carnitine has been found to decrease the expression of SARS-CoV-2 receptors ACE2 and proteases TMPRSS2 and Furin in human pulmonary epithelial cells by upregulating hepatocyte nuclear factor 4 alpha (HNF4-α). Furthermore, it inhibits NF-κB expression and downregulates NADPH oxidase-1 (NOX-1) and -2, thereby reducing hyperactivated inflammatory pathways in COVID-19. In COVID-19, L-carnitine administration enhances CPT-1 expression in lymphocytes, correlating with an increased spare respiratory capacity and resilience of memory T cells post-infection [34]. Talebi et al. [35] conducted a study on the administration of 3000 mg of oral L-carnitine daily in three divided doses over five days in patients with mild-to-moderate COVID-19, alongside standard treatments. The intervention group exhibited significant improvements in clinical parameters, including increased oxygen saturation (*p* = 0.039) and reduced inflammatory markers such as the erythrocyte sedimentation rate (ESR) (*p* = 0.021), CRP (*p* = 0.009), mean hemoglobin (Hb) (*p* = 0.026), alkaline phosphatase (ALP) (*p* = 0.010), lactate dehydrogenase (LDH) (*p* = 0.002), and creatine phosphokinase (CPK) (*p* = 0.019) compared to the control group. Importantly, six patients (14%) in the control group died from COVID-19 complications, while all patients in the intervention group survived (*p* = 0.030).

## 3. Functional Antioxidants

### 3.1. Bromelain

Bromelain is a complex mixture of thiol proteolytic enzymes predominantly derived from the pineapple plant, encompassing a diverse array of endopeptidases, phosphatases, glucosidases, peroxidases, cellulases, glycoproteins, carbohydrates, and protease inhibitors. These enzymes are classified within the cysteine protease family and exhibit distinct properties attributed to the presence of sulfhydryl groups in their structure. Bromelain exhibits therapeutic effects through a multifaceted mechanism that includes its proteolytic, anti-inflammatory, immunomodulatory, fibrinolytic, and antioxidant activities and its influence on cell signaling pathways. Although bromelain is widely recognized for its role in aiding protein digestion, its primary functions are more closely associated with its antioxidant properties, which include scavenging free radicals and ROS to safeguard cells against oxidative damage [36]. Bromelain exhibits immunomodulatory properties that enhance immune function by promoting the activity of immune cells, including macrophages, NK cells, and lymphocytes, through the activation of phosphoinositide 3-kinase/protein kinase B (PI3K/Akt) and mitogen-activated protein kinase (MAPK) pathways. It inhibits mitogenesis, apoptosis, and cytokine formation by preventing CD4+ T cell activation during inflammation via the suppression of the proto-oncogene serine/threonine-protein kinase (Raf-1)/extracellular-regulated-kinase-2 (ERK-2) pathway. Additionally, bromelain helps maintain a balanced cytokine profile by modulating the production of pro-inflammatory and anti-inflammatory cytokines, which are crucial for immune responses. In the context of SARS-CoV-2 infection, it may attenuate the cytokine storm by inhibiting IL-6 and TNF-α, thus minimizing tissue damage and slowing inflammation progression. By downregulating inducible nitric oxide synthase (iNOS) and cyclooxygenase-2 (COX-2) through the Akt phosphorylation, bromelain limits the synthesis of the inflammatory mediator such as bradykinin and prostaglandin E2 (PGE2), helping restore antioxidant balance, limiting tissue damage. Tissue integrity is preserved through the reduction in advanced glycation end-product (AGE) receptors, which occurs via the degradation of its receptors. Vascular changes related to SARS-CoV-2 are balanced by promoting fibrinolysis through an enhanced plasmin concentration and regulating angiogenic biomarkers like vascular endothelial growth factor (VEGF) and matrix metalloproteinases (MMPs) for vascular remodeling and tissue repair. Lastly, bromelain causes an anticoagulant effect by inhibiting platelet aggregation and suppressing pro-inflammatory cytokines by enhancing the plasmin concentration, vital in mitigating thrombosis associated with increased kinins during COVID-19 infection [37]. In their study, Jahangirifard et al. [38] examined the effect of a 600 mg daily dose of bromelain administered orally for 5 days in patients recovering from COVID-19 pneumonia, given its antioxidant and anti-inflammatory properties. The results indicated that bromelain significantly influenced inflammatory parameters, with the clinical assessments of the SpO2, respiratory rate (RR), and heart rate (HR) showing notable differences between the treatment and control groups (*p* < 0.05). Furthermore, laboratory analyses revealed significant alterations in inflammatory markers, including CRP, ESR, and LDH (*p* < 0.05). Immunological factors such as the white blood cell (WBC) count and lymphocyte levels, along with renal (blood urea nitrogen [BUN]) and liver function tests (aspartate aminotransferase [AST] and alanine aminotransferase [ALT]), also demonstrated significant differences (*p* < 0.05).

### 3.2. N-Acetylcysteine

NAC is a thiol, mucolytic agent, and precursor of L-cysteine and GSH. It acts as a scavenger of ROS like hydroxyl radicals (OH^−^) and H_2_O_2_, influencing processes such as cell adhesion, oxidative stress, smooth muscle cell proliferation, and the stability of atherosclerotic plaques. NAC reduces lung inflammation, fibrosis, and smoking-related changes. In endothelial function, NAC lowers ROS levels, increasing nitric oxide bioavailability and suppressing inflammatory cytokines (TNF-α, IL-1, vascular cell adhesion molecule-1 [VCAM-1], and E-selectin) through NF-kB inhibition [39]. In respiratory systems, NAC has anti-inflammatory and antioxidant effects, inhibiting TNF-α-induced NF-kB activation and interleukin-8 production [40]. It also protects against cigarette smoke-induced lung pathology by inhibiting TGF-β and reduces TNF-α-induced activation of MAPK, aiding in lung injury protection [41,42]. Traditionally used as an antidote for paracetamol overdose and as a mucolytic agent, NAC has shown promise in enhancing immune function, inhibiting viral replication, and reducing inflammatory responses in acute viral respiratory infections like influenza and ARDS [39]. Its potential role in mitigating COVID-19-induced inflammation and cytokine storms has been explored, with studies suggesting NAC may suppress viral replication and enhance immune responses [43]. NAC inhibits SARS-CoV-2 cell infection by diminishing the affinity of the ACE2 receptor for the virus’s S protein through the action of its thiol groups. It may also impair viral protease activity by binding to Cys-145, thereby inhibiting viral replication. Additionally, NAC can activate Toll-like receptor 7 (TLR7) and mitochondrial antiviral signaling (MAVS), enhancing IFN-1 production that suppresses SARS-CoV-2 replication. Furthermore, it promotes the production of endogenous hydrogen sulfide (H_2_S), contributing to antiviral responses [44]. A pilot study comparing intravenous NAC (40 mg/kg/day for three days) to a placebo in mild-to-moderate COVID-19-associated ARDS patients showed no significant differences in 28-day mortality, ICU access, or length of hospital stay, despite better outcomes in the NAC group [45].

### 3.3. Melatonin

Melatonin, a neurohormone derived from the essential amino acid tryptophan, is synthesized in mitochondria through two enzymes: arylalkylamine N-acetyltransferase (AANAT) and acetylserotonin-O-methyltransferase (ASMT), with AANAT being rate-limiting [46]. While primarily produced in the pineal gland and retina, melatonin can be synthesized in various tissues, including the gastrointestinal tract, bone marrow, lymphocytes, skin, lungs, and brain. Its levels in the pineal gland and blood fluctuate in a circadian pattern, regulated by the suprachiasmatic nucleus in response to the light cycle, peaking at night and remaining low during the day. Melatonin plays key roles in sleep regulation, blood pressure control, mitochondrial maintenance, and antioxidant and antiviral effects [47]. Melatonin also has notable immune-modulatory effects, enhancing the movement of NK cells and other immune cells [48]. In cases of uncontrolled inflammation, it reduces neutrophil infiltration and mitigates tissue damage in conditions like acute lung injury and pancreatitis. Melatonin inhibits the adhesion and migration of immune cells, particularly by downregulating leukotriene B4-induced adhesiveness in endothelial cells and reducing IL-1β levels, preserving vascular integrity [49]. Given its anti-inflammatory properties, melatonin has shown promise in limiting viral diseases such as COVID-19. During infection, it helps maintain lung integrity by reducing proteolytic enzymes, ROS, and reactive nitrogen species (RNS), preventing DNA damage and oxidative stress in the alveolar sacs. Melatonin has been shown to inhibit the binding of ACE2 receptors to SARS-CoV-2, thereby obstructing viral entry and replication. This action occurs through calmodulin’s blockade and viral chymotrypsin-like protease activity suppression. Furthermore, melatonin activates sirtuin-1 (SIRT-1), which plays a crucial role in preventing the formation of hyperinflammatory macrophages, thereby enhancing its protective efficacy against viral proliferation within host cells. The compound also inhibits the NOD-like receptor family pyrin domain containing 3 (NLRP3) inflammasome, resulting in the reduction in pro-inflammatory cytokines such as IL-18 and IL-1β, thus exerting significant anti-inflammatory effects. Additionally, melatonin inhibits NF-κB signaling pathways, downregulates iNOS and COX-2, and prevents TLR4 activation, collectively leading to a reduction in cytokine storm levels characterized by elevated TNF-α, IL-1β, IL-6, and IL-8 [50,51]. In a randomized clinical trial by Farnoosh et al. [52], 24 hospitalized patients with mild to moderate COVID-19 were given 3 mg of melatonin three times daily for 14 days, in addition to standard care. The results showed significant improvements in clinical symptoms, inflammatory biomarkers like CRP, pulmonary involvement, and hospitalization length compared to those of the control group. The study concluded that melatonin could serve as an effective adjuvant therapy, reducing oxidative stress and enhancing antioxidant enzyme activity.

## 4. Vitamins

### 4.1. Vitamin A

Vitamin A comprises a group of fat-soluble retinoids sharing a four-isoprenoid-unit structure and function, including retinol, retinal, and retinyl esters. Animals cannot synthesize vitamin A de novo and must obtain it from their diet, primarily from animal sources in the form of retinol and its close derivatives, or from plant sources such as carrots and other dark-colored fruits in the form of provitamin carotenoids like beta-carotene. Retinol is absorbed from the digestive tract while beta-carotene is taken up by enterocytes via the scavenger receptor B1 transporter. Vitamin A is stored as retinyl esters in liver stellate cells and can be oxidized to retinal and retinoic acid in tissues. While vitamin A is primarily linked to retinol, the predominant retinoid in the human body, the primary biologically active forms are the oxidized derivatives 11-cis-retinal and all-trans-retinoic acid (ATRA). Retinoids are classified into four generations, starting from naturally occurring first-generation forms like retinol and tretinoin to synthesized second-generation retinoids like etretinate, third-generation compounds such as adapalene, and the fourth generation, represented solely by trifarotene. Vitamin A performs diverse functions in various tissues through its active forms, such as retinol as a cofactor in enzymatic processes, 11-cis-retinal in vision, and ATRA in the regulation of gene expression via nuclear receptor binding [53]. Retinoic acid exerts its immunomodulatory effects through interactions with nuclear receptors, including the retinoic acid receptor (RAR), retinoid receptor X (RXR), and peroxisome proliferator-activated receptor-β (PPAR-β), thereby regulating the transcription of various genes related to cytokines, chemokines, and integrins, as well as those involved in lipid metabolism and glucose homeostasis [54]. Vitamin A is crucial in various physiological functions, including vision, growth, reproduction, hematopoiesis, immunity, and cellular integrity. Vitamin A is essential for the immune system, with deficiencies linked to increased infection susceptibility, especially in children. It is known as the “anti-infective vitamin” for its role in recovery rather than prevention. Research by Tepasse et al. [55] indicates that hospitalized COVID-19 patients exhibit significantly lower vitamin A levels, particularly those that are critically ill, with levels below 0.2 mg/L strongly associated with an increased risk of developing ARDS (*p* = 0.048) and mortality (*p* = 0.042). Vitamin A is particularly important in infectious diseases, especially pulmonary infections, as it supports the development of normal lung tissue and aids recovery post-infection. It plays a key role in regulating immune functions, affecting innate and adaptive immune responses. Specifically, vitamin A is essential for T cell, T helper cell, and B cell growth, with deficiency impairing antibody responses and disrupting innate immunity by inhibiting mucosal epithelium regeneration and reducing immune cell function. Moreover, vitamin A promotes T lymphocyte proliferation and differentiation, particularly in regulatory T cells, and contributes to lung regeneration and improved antibody production in vaccine responses. Recent research suggests that vitamin A may serve as a viable therapeutic target for COVID-19 by modulating inflammatory responses and oxidative stress, thereby potentially influencing the entry of SARS-CoV-2. It was proposed that the inhibition of SARS-CoV-2 cell entry is mediated by the activation of retinoic acid-inducible gene I (RIG-I) and melanoma differentiation-associated protein 5 (MDA5). These cytosolic receptors play a crucial role in suppressing viral infections by recognizing double-stranded RNA produced by viral pathogens and inducing alpha/beta interferon production via the NF-kB pathway [56]. Vitamin A has been shown to inhibit COVID-19 primarily by suppressing the MAPK1 signaling pathway, which plays a crucial role in linking extracellular stimuli to intracellular responses. Additionally, it affects the epidermal growth factor receptor (EGFR), a molecule vital for regulating cell growth, division, differentiation, survival, and oncogenesis. The induction of IL-10, a significant anti-inflammatory cytokine, alongside intracellular adhesion molecule-1 (ICAM-1), a receptor with chemoattractant functions, contributes to reducing the severity of COVID-19 pneumonia. Furthermore, the modulation of catalase, an antioxidant enzyme, and protein kinase C-β (PRKCB), a key regulator of B cells, is essential for effectively managing COVID-19 infection [57]. Building on these promising foundations, Somi et al. [58] administered intramuscular vitamin A to 15 hospitalized patients with mild-to-moderate COVID-19 at a dosage of 50,000 IU/day for a maximum duration of two weeks (average 7.33 ± 2.31 days). This treatment was provided in conjunction with standard therapies, which included corticosteroids, antivirals, and antibiotics. The authors observed no significant differences between the vitamin A and placebo groups regarding ICU admission rates, length of hospital stay, clinical response, respiratory support, and mortality.

### 4.2. Vitamin B_12_

Vitamin B_12_, known as cobalamin, consists of a corrin ring with cobalt at its core and exists in multiple forms, including cyanocobalamin, methylcobalamin, deoxyadenosylcobalamin, and hydroxycobalamin. Its primary dietary sources include liver, meat, dairy products, eggs, and certain fish, while plant-based foods lack vitamin B_12_, resulting in higher deficiency rates among vegetarians and vegans, who may require supplementation. Vitamin B_12_ is released from dietary proteins in the stomach, binds to haptocorrin, and is absorbed in the proximal ileum with an intrinsic factor. Circulating B_12_ primarily attaches to transcobalamin for cellular delivery, modulating immune responses and inflammation. This water-soluble vitamin plays crucial roles in metabolic processes, cardiovascular and circulatory systems, immune system control, and antiviral activities. Vitamin B_12_ is involved in repairing tissue damage and compensating for diminished hepatic storage during viral hepatitis. It is also crucial for DNA synthesis and metabolic pathways involving lipids, carbohydrates, and proteins. It functions as a cofactor for methionine synthase (MS) and methylmalonyl-CoA mutase, both essential enzymes in methyl biosynthesis. This process involves the conversion of homocysteine to methionine, followed by the transformation of methionine into S-adenosylmethionine (SAM). Methyl cobalamin is essential for the production of methionine and S-adenosyl methionine, impacting myelin integrity, neurological function, and red blood cell synthesis. Adenosyl cobalamin, also known as coenzyme-B_12_, functions as a cofactor in redox reactions, engaging in the generation of reactive free radicals [59]. Vitamin B_12_ has been identified as an immunomodulatory agent that can enhance immune responses by promoting the proliferation of CD8+ T cells and natural killer T cells. Furthermore, vitamin B_12_ exhibits notable antioxidant properties, which include increasing the bioavailability of reduced GSH and facilitating the synthesis of oxidized GSH. It functions as a negative regulator of NF-κB through nitric oxide modulation mechanisms. This biological activity positions vitamin B_12_ as a potential adjunctive therapy in managing viral infections, including hepatitis, human immunodeficiency virus (HIV), and Norovirus, with emerging evidence suggesting effects in COVID-19. Specific SARS-CoV-2 proteins like ORF3a and ORF7a can enhance NF-κB levels, worsening COVID-19-related lung inflammation, while Vitamin B_12_ may reduce NF-κB activity and pro-inflammatory cytokine expression, potentially lowering the need for mechanical ventilation. Supplementation with vitamin B_12_ in COVID-19 patients may alleviate symptoms indicative of deficiency, alleviate pain and depressive symptoms, enhance cognitive function, and mitigate cellular damage through its anti-inflammatory and antioxidant actions. Moreover, transcobalamins have been implicated in suppressing systemic inflammation through their regulatory effects on various cytokines, such as IL-6, as well as their influence on growth factors and other anti-inflammatory mediators. The antiviral properties of vitamin B_12_ are associated with its ability to obstruct the entry of SARS-CoV-2 into host cells by binding to specific viral cell receptors, including ACE2, furin, DPP-4, and human aminopeptidase N (hAPN). Additionally, through the inactivation of RdRp and 3CLpro, vitamin B_12_ diminishes viral replication [60]. In a study by Erfani et al. [61], the daily administration of 1000 mg of vitamin B_12_ over 7 days was given to hospitalized patients diagnosed with COVID-19. The results indicated notable improvements in various biomarkers, including the CRP, LDH, creatinine, ferritin, and ALT levels in the treatment group, in comparison to the control group, which exhibited a higher rate of ICU admissions. Nonetheless, it is important to note that the changes observed and the admission rates did not attain statistical significance.

### 4.3. Vitamin C

Vitamin C, or ascorbic acid, is an essential water-soluble vitamin known for its antioxidant properties and role in immune function. Since humans cannot synthesize it, it must be obtained through diet. Skeletal muscle serves as the primary reservoir for vitamin C, and inadequate intake leads to rapid depletion [62]. As an antioxidant, it protects against oxidative damage, maintains the skin’s epithelial barrier, and supports immune cells by reducing oxidative stress, promoting apoptosis, and inhibiting necrosis [63]. It also modulates inflammatory responses by downregulating NF-kB and reducing pro-inflammatory cytokines. In COVID-19 patients, vitamin C reduces inflammatory mediators, excessive nitrate production, and oxidative stress [64]. However, high doses may have pro-oxidant effects by depleting ROS scavengers like GSH and NADPH, potentially increasing DNA damage [65]. Vitamin C also plays a role in antiviral defense, particularly in enhancing immune responses and improving phagocyte migration. It shifts immune responses from Th2 to Th1 and induces Th17 polarization in murine models [66]. While its influence on antibody production is debated, adequate levels are crucial for NK cell function. Vitamin C plays a critical role in regulating inflammatory cytokines, specifically by enhancing the levels of Th1 cytokines such as TNF-α, IFN-γ, and IL-12, while simultaneously reducing the levels of Th2 cytokines, particularly IL-4 [67]. Recent studies on intravenous (IV) vitamin C suggest potential benefits in treating pneumonia and COVID-19, especially in ICU patients, as IV administration can achieve much higher plasma concentrations than oral intake [68]. Vitamin C may help balance inflammatory responses in ARDS, although the results are mixed regarding its effects on ventilation duration and pro-inflammatory biomarkers. Furthermore, the thrombotic complications observed in COVID-19 patients may be mitigated by inhibiting the pathways involved in the formation of Neutrophil Extracellular Traps (NETs). Vitamin C may exert a direct antiviral effect in the context of COVID-19 by reducing the expression of ACE2, furin, and cathepsin L, which are key enzymes involved in the entry of SARS-CoV-2 into human cells. It has been hypothesized that vitamin C may also interfere with viral replication by inhibiting 3CLpro, a critical protease in the SARS-CoV-2 life cycle, as well as the activity of RdRP, which is essential for viral protein synthesis [69]. One study showed a reduced ventilation time in patients receiving high-dose vitamin C, but a meta-analysis showed no significant improvement [70,71]. An Iranian study reported that administering 8 g of IV vitamin C over 5 days improved the oxygen saturation and decreased the respiratory rate in patients with moderate COVID-19 pneumonia. Additionally, radiological lung involvement showed improvement compared to the control group [72]. The WHO recognizes vitamin C’s immunomodulatory role, and ongoing trials are exploring its potential benefits in managing COVID-19, especially in critically ill patients [73].

### 4.4. Vitamin D

Vitamin D, or calciferol, is a fat-soluble vitamin obtained through supplements or synthesized by the body when exposed to ultraviolet rays. To become active, it undergoes two hydroxylation steps: the first is in the liver, producing 25-hydroxyvitamin D [25(OH)D], and the second is in the kidney, producing 1,25(OH)2D [74]. The latter binds strongly to the vitamin D receptor (VDR), influencing gene expression across various biological processes. Vitamin D plays a critical role in regulating calcium (Ca) and phosphate metabolism, maintaining bone health, and influencing conditions such as cancer, cardiovascular disease, infections, and autoimmune disorders. Numerous studies have linked circulating 25(OH)D levels to various health outcomes [75]. Vitamin D exhibits its action as a free radical scavenger primarily through the suppression of NOX activity, coupled with an enhancement in the activity of catalase and SOD. Beyond bone health, vitamin D is essential for immune system regulation, particularly in respiratory infections. Research from past coronavirus pandemics suggests that vitamin D supplementation may improve immune responses and alleviate symptoms like cough and loss of taste (ageusia) in COVID-19 [76]. Vitamin D may enhance antiviral effects by interacting with its receptor, improving IFN signaling, and promoting autophagy by acidifying endolysosomes [77]. The loss of taste and smell during respiratory infections may be due to the excessive activation of immune pathways. It is hypothesized that vitamin D may help restore these senses by reducing inflammation and supporting the taste and smell systems [78]. Additionally, vitamin D’s neuroprotective effects, through the regulation of neurotrophins, may contribute to this restoration [79]. Furthermore, vitamin D modulates the immune response by regulating inflammatory cytokine production (TNF-α, IL-1, IL-6, and IL-8) and influencing the NLRP3 inflammasome. Moreover, vitamin D inhibits the cytokine storm by switching the pro-inflammatory Th1 and Th17 to the anti-inflammatory Th2 and Treg response, inhibiting the production of pro-inflammatory cytokines from Th1 cells such as TNF-α and INF-γ. Vitamin D functions as a dual protector against COVID-19 by promoting antiviral mechanisms and modulating inflammatory responses. It enhances macrophage production of antimicrobial proteins such as β-defensin 2 and cathelicidin, which inhibit viral replication and facilitate autophagy. Additionally, vitamin D induces the expression of nuclear factor of kappa light polypeptide gene enhancer in B-cell inhibitor α (IkBα), inhibiting NF-kB and reducing inflammatory gene expression [80]. Several studies have linked low vitamin D levels with severe COVID-19 outcomes, including intensive care unit (ICU) admission and death [81,82]. Some trials suggest that high-dose vitamin D supplementation may prevent ICU admission, reduce recovery time, and lower inflammatory markers. The potential benefits are thought to stem from vitamin D’s ability to increase angiotensin-converting enzyme (ACE2) receptor expression, which facilitates SARS-CoV-2 entry, or its promotion of antimicrobial peptides that reduce inflammation in the respiratory epithelium [83,84]. Vitamin D may exert antiviral effects by modulating the ACE2 pathway, decreasing the ACE concentration and the ACE/ACE2 ratio while promoting ACE2 levels. Sufficient vitamin D suppresses the renin-angiotensin system (RAS), increases ACE2 expression, and converts Angiotensin-II to Angiotensin (1–7), promoting vasodilatory, anti-inflammatory, and anti-thrombotic effects. Soluble ACE2 binds and neutralizes circulating SARS-CoV-2. Additionally, ACE inhibitors and angiotensin receptor blockers (ARBs) reduce Angiotensin-II production and its interaction with the Angiotensin-II type-1 receptor, further diminishing SARS-CoV-2’s harmful effects [85]. A study by Sabico et al. [86] found that a 2-week regimen of 5000 IU daily vitamin D3 supplementation significantly shortened recovery times for symptoms like cough and ageusia in COVID-19 patients with low vitamin D status compared to the standard 1000 IU dose.

### 4.5. Vitamin E

Vitamin E is a fat-soluble antioxidant primarily found in nuts, seeds, and tropical fruits. It protects polyunsaturated fatty acids (PUFAs) in cell membranes from oxidation, regulating the production of ROS and RNS, thereby preventing lipid peroxidation and maintaining membrane integrity. Additionally, it mitigates UV radiation damage, highlighting its protective role against skin diseases. Vitamin E enhances the dephosphorylation of protein kinase C-α (PKC-α) via the activation of protein phosphatase 2A, influencing various cellular functions, including the suppression of platelet aggregation and reduced macrophage proliferation. Vitamin E consists of eight isoforms, namely, tocopherols and tocotrienols, with α-tocopherol recognized as the most effective antioxidant, demonstrating an affinity for peroxyl radicals that is approximately 1000 times greater than that for PUFAs. The oxidized form, tocopheryl radical, can be regenerated by hydrogen donors such as vitamin C and coenzyme Q, thereby justifying their synergistic enhancement of antioxidant activity in infectious diseases when used together [87]. Vitamin E is involved in the functioning of the immune system, showing immunomodulatory and anti-inflammatory effects. It enhances lymphocyte activity and the NK cell response, influencing the relationship between dendritic cells and CD4+ T cells. This vitamin enhances NK cell function by modulating NO levels, leading to decreased NO production, COX-2 inhibition, and the downregulation of PGE2. Supplementation has been shown to improve cell-mediated immunity through various pathways, including increased Th1 cell activity and IL-2 production. Vitamin E influences inflammatory responses in various tissues, including the lungs, by scavenging oxidative stress and modulating eicosanoid pathways and prostaglandin synthesis. The immunoregulatory function of vitamin E is clinically relevant, as it may lower susceptibility to viral infections and reduce the risk of respiratory diseases [88]. An inverse relationship between vitamin E levels and plasma lipoperoxidase in ARDS patients suggests that vitamin E deficiency increases lipid peroxidation, while elevated vitamin E in the immune cells of COVID-19 patients may protect against oxidative damage, highlighting its potential as an antioxidant in mitigating oxidative stress associated with SARS-CoV-2 pathogenesis. An additional antiviral effect is achieved by inhibiting viral replication, which occurs by interfering with the activity of RdRp and 3C-like protease (3CLpro). The effectiveness of vitamin E supplementation in SARS-CoV-2 infection remains unclear, although a combination with vitamin C may provide antioxidant benefits, particularly against cardiac complications. In light of the established antioxidant and immunomodulatory properties of vitamins E and C, Hakamifard et al. [89] conducted a study to examine the effects of co-administering both vitamins in addition to the national standard treatment regimen (hydroxychloroquine) for patients hospitalized with non-severe COVID-19 pneumonia. Despite high expectations, the combination of low-dose oral vitamin C (1000 mg daily) and high-dose vitamin E (400 IU daily) shows no beneficial effect in patients with COVID-19.

## 5. Polyphenols

### 5.1. Curcumin

Curcumin, a polyphenolic compound from *Curcuma longa*, has attracted significant scientific attention for its antioxidant, anticancer, and anti-inflammatory effects [90]. It modulates pro-inflammatory cytokines (IL-1, IL-6, and TNF-α) through the NRF-2 pathway, which plays a key role in lung inflammation [91]. Curcumin inhibits the production of cytokines and chemokines, such as MMP family, monocyte chemoattractant protein-1 (MCP-1), macrophage inflammatory protein-1 (MIPI-1), stromal cell-derived factor-1 (SDF-1), and CXCL, and downregulates inflammatory pathways like MAPKs, Jun N-terminal kinase (JNK), and NF-kB [92]. Its antioxidant properties include inhibiting the production of carcinogenic ROS, such as O_2_^−^, OH^−^, and H_2_O_2_ [93]. The enzyme NADPH: quinone oxidoreductase 1 (NQO1) plays a critical role in antioxidative defense and is regulated by NRF-2 [94]. In COVID-19, curcumin’s potential antiviral effects have been explored, showing promise in modulating inflammation and immune responses, possibly reducing viral replication, pulmonary edema, and fibrosis [95]. Curcumin has demonstrated direct antiviral effects by inhibiting viral replication through the disruption of the virion structure. Specifically, it inhibits the dimerization of the SARS-CoV-2 nucleocapsid C-terminal domain and downregulates the activities of the viral complexes PLpro and RdRp [96]. A systematic review found that curcumin supplementation alleviates symptoms, reduces hospitalization time, and lowers mortality by counteracting cytokine storms and restoring inflammatory balance [97]. However, curcumin’s bioavailability remains a challenge, prompting the development of nanocurcumin, a formulation using biodegradable nanoparticles to enhance solubility and stability [98]. A recent Iranian study investigated the effect of 160 mg of daily nanocurcumin in hospitalized COVID-19 pneumonia patients [99]. This formulation significantly improved curcumin’s bioavailability, leading to faster symptom relief (cough, fatigue, and myalgia) and reduced oxygen demand, oxygen use, and respiratory rates compared to a placebo. The patients who received nanocurcumin also had a greater increase in oxygen saturation at discharge.

### 5.2. Quercetin

Quercetin, a natural flavonoid found in foods like green leafy plants, grapes, apples, and onions, has gained attention for its role in combating viral infections, particularly SARS-CoV-2. Known chemically as 3,3′,4′,5,7-pentahydroxyflavone, quercetin and other polyphenols act as antioxidants, scavenging ROS and free radicals, while promoting phase II detoxification enzymes. Its antioxidant properties are mediated through the inhibition of enzymes like xanthine oxidase (XO) and NOX, which are involved in the production of ROS and RNS [100]. The immunomodulatory effects of quercetin are characterized by the promotion of IL-10) release, alongside the reduction in TNF-α and IL-1β levels [101]. Research shows that quercetin can affect viral entry and boost immune response regulation, influencing over 85% of SARS-CoV-2 structural proteins. Its primary mechanisms include inhibiting viral entry and replication, as well as suppressing NLRP3 inflammasome activation, contributing to its anti-inflammatory properties [102]. Additionally, quercetin may modulate the acid sphingomyelinase/ceramide system, which is crucial for virus internalization in respiratory cells [103]. Clinical trials suggest that inhibiting this pathway could reduce intubation and mortality risks in COVID-19 patients. Molecular docking studies reveal that quercetin binds to several SARS-CoV-2 proteins, including the S protein, 3CLpro, PLpro, and RdRp, as well as key cellular receptors like ACE2 and TMPRSS2 [104]. By binding to ACE2, quercetin helps prevent syncytia formation [105]. Inhibiting furin and TMPRSS2 also blocks SARS-CoV-2 endoproteolysis [106]. Notably, quercetin may disrupt membrane enzymes by intercalating into the lipid bilayer, impeding S2 protein binding to furin. Quercetin also non-competitively inhibits the activities of RNA helicase (RHA) and 5’-triphosphatase, both of which are associated with NSP13, a crucial component of SARS-CoV-2’s viral replication functions [107]. Moreover, quercetin’s antioxidant and anti-inflammatory effects help mitigate oxidative stress and inflammation, both critical in COVID-19 pathophysiology [108]. A clinical trial by Shohan et al. [109] evaluated 1000 mg of quercetin daily in patients with SARS-CoV-2 pneumonia, in addition to antiviral therapy. The results showed that quercetin significantly reduced the hospitalization time and serum levels of ALP, CRP, and LDH. The patients who took quercetin also had higher hemoglobin levels and improved respiratory rates, suggesting its potential benefits. Further studies are needed to assess its impact on mortality and ICU admissions.

### 5.3. Resveratrol

Resveratrol, a non-flavonoid bioactive polyphenol, exhibits notable anti-inflammatory and antiviral properties, particularly against respiratory viruses like influenza A, respiratory syncytial virus (RSV), human metapneumovirus, MERS-CoV, and SARS-CoV-2. It inhibits viral replication and modulates inflammatory responses by targeting pathways such as NF-κB, IL-17, TNF-α, and ERK/MAPK. Resveratrol may also downregulate fibroblastic growth factor (FGF-2) signaling, which is linked to virus-induced apoptosis [110]. Its antioxidant effects are primarily mediated by NRF-2 activation, which boosts the transcription of antioxidant genes and key enzymes like endothelial nitric oxide synthase (eNOS), NQO1, and GSH S-transferase. Additionally, resveratrol inhibits NOX and PPAR-γ, which are involved in ROS production and severe COVID-19 outcomes, while increasing NO bioavailability, supporting its vasodilatory and antiplatelet effects. It may protect the endothelial barrier by reducing thrombosis markers, possibly through SIRT-1 pathway [111]. Resveratrol has been identified as an inhibitor of SARS-CoV-2 infection, primarily through its capacity to reduce the expression of the N protein, leading to decreased viral production and improved cellular survival. Furthermore, resveratrol appears to disrupt the viral endocytosis process by inhibiting the binding of the viral S protein to the ACE2 receptor. Additionally, it may hinder viral replication by inactivating key enzymes, including SARS-CoV-2 RdRp, PLpro, and 3CLpro [112]. However, its clinical application has been limited by low bioavailability. A phase II multicentric clinical trial (TAEROVID-19) [113] tested resveratrol nebulization in hospitalized COVID-19 pneumonia patients. Forty-three patients received standard care plus an aerosol formulation containing 100 mg of Taurisolo^®^ (MBMed: Turin, Italy) and 4.75 mg of Polygonum cuspidatum extract, primarily resveratrol, administered three times daily for 14 days. After a median follow-up of 10.5 days, only one patient (2.33%) required ICU admission, indicating a lower risk of clinical worsening. The treatment led to significant improvements, including a rise in the P/F ratio (from 292 to 310, *p* = 0.033) and oxygen saturation (from 95.8% to 97.1%, *p* < 0.001). Inflammatory markers, such as CRP (from 8.8 to 0.5, *p* < 0.001), IL-6 (from 22 to 4.3, *p* < 0.001), and fibrinogen (from 585 to 377, *p* < 0.001), decreased significantly. Although the decrease in the viral load was not statistically significant, the trial suggests that resveratrol aerosol therapy may reduce COVID-19 symptoms and enhance recovery in non-hospitalized patients. Further research is needed to confirm these findings.

## 6. Trace Elements

### 6.1. Zinc

Zn is a critical micronutrient that supports the growth, development, and maintenance of immune barriers, such as skin and mucous membranes. It plays a significant role in antiviral action and the clinical course of viral infections by reducing oxidative stress and inflammation. Zn is essential for the development of immune cells, particularly T lymphocytes, and deficiencies can impair immune responses, increasing the risk of severe outcomes from infections like pneumonia. Additionally, Zn aids in macrophage function and the synthesis of cytokines such as IFN, IL-2, and IL-12, activating T cytotoxic cells and NK cells and influencing immune responses against viral and bacterial pathogens. Zn deficiency disrupts IL-10 production, affecting Th1 responses and macrophage activities, while Zn supplementation has been shown to reduce TNF-α and IL-1 levels in healthy individuals. Specific to SARS-CoV-2 infection, Zn may inhibit the reproduction and proliferation of virus by preventing RNA synthesis and modulating cytokine mRNA levels, potentially preventing the progression of COVID-19 and cytokine storms. Zn displays antiviral properties by inhibiting virus fixation, infection, and coating while disrupting the proteolytic processing of viral polyproteins. Additionally, high concentrations of Zn can alter the structure of viral proteases and prevent the fusion of viral and host cellular membranes, thereby impeding viral infection. Recent studies suggest that higher Zn levels could suppress ACE2 expression, potentially reducing receptor interaction with SARS-CoV-2, while lower levels may enhance this interaction, indicating a protective role against COVID-19 [114]. Zn cations, particularly when combined with the ionophore pyrithione, inhibit SARS-CoV-2 RdRp activity, thereby reducing viral replication and disrupting polyprotein processing. Ionophores such as hydroxychloroquine, chloroquine, pyrrolidine dithiocarbamate, and pyrithione enhance Zn’s antiviral effects by facilitating its cellular influx, thereby inhibiting the replication of several RNA viruses, including SARS-CoV-2, influenza, and RSV [115]. Despite a previous study by Frontera et al. [116] indicating that the co-administration of Zn with an ionophore, like chloroquine, enhances intracellular Zn uptake and significantly decreases COVID-19 hospitalization and mortality rates, there remains a considerable deficiency in research exploring the effects of Zn when employed alone. In this context, Yao et al. [117] examined the effects of the daily administration of 440 mg (equivalent to 100 mg of elemental Zn) of Zn sulfate in a cohort of 40 patients hospitalized with mild COVID-19. The study demonstrated the absence of a causal relationship between Zn supplementation and both survival rates and the length of hospitalization among COVID-19 patients.

### 6.2. Magnesium

Mg is the second most abundant cation in the body’s cells and is essential for numerous physiological functions, acting as a cofactor for over 600 enzymes. Regular dietary intake is necessary to prevent deficiency, and Mg is commonly found in seeds, legumes, nuts, whole grains, certain fruits, and cocoa. Mg is vital for various metabolic and biochemical processes, contributing to bone development, neuromuscular function, signal transmission, energy production, and the metabolism of glucose, lipids, and proteins. It also stabilizes DNA and RNA and regulates cell growth and specialization. Furthermore, Mg plays a significant role in the functions of both the innate and adaptive immune systems by balancing inflammation and maintaining endothelial function, which may offer protective benefits against COVID-19. It stabilizes mastocyte membranes, regulates neutrophil and macrophage activity, and inhibits the TLR/NF-κB pathway. Additionally, Mg modulates the cytotoxic activities of NK cells and CD8+ T lymphocytes. In lymphocytes, Mg regulates the levels of Inositol Triphosphate and diacylglycerol, which are essential second messengers that become active upon the activation of B and T cell receptors. Moreover, Mg plays a crucial role in defending the body against viral infections, as it is necessary to maintain sufficient levels of intracellular Mg for the cytotoxic function of T lymphocytes and NK cells. Mg exerts an inhibitory effect on inflammatory mediators, including chemokines like MIPI-2; cytokines such as TNFα, IL-1, and IL-6; PGE2; and COX-2 in lung tissue, potentially by modulating L-type Ca channels. Furthermore, considering the critical role of the Mg status in vitamin D metabolism as a cofactor in various metabolic pathways, many of Mg’s immunomodulatory effects can be attributed to its relationship with vitamin D. Low Mg levels are associated with pro-inflammatory states, increased endothelial thrombogenicity, and chronic low-grade inflammation, which may exacerbate virus-induced inflammation and endothelial dysfunction in COVID-19 patients. Hypomagnesemia, a prevalent deficiency in critically ill patients, is linked to infection severity, extended ICU stays, and increased mortality, indicating its association with poor COVID-19 outcomes [118]. In addition, Mg may inhibit SARS-CoV-2 entry by directly affecting TMPRSS2 through methylation changes in its gene and indirectly by modulating platelet-aggregating factor (PAF) synthesis via the inhibition of lyso-PAF-acetyltransferase. PAF, primarily synthesized in platelets, exhibits pro-inflammatory properties, induces bronchoconstriction, and plays a role in modulating ACE and ACE2 activity [119]. Mg supplementation may enhance the respiratory function in COVID-19 patients by maintaining airway smooth muscle tone, promoting bronchodilation, and reducing airway inflammation through its influence on Ca homeostasis and ion channels. Mg supplementation may positively impact neurological development and mental well-being by reducing depression and anxiety, particularly in COVID-19 patients. This effect is likely due to Mg’s neuroprotective properties and critical role in neurotransmitter regulation, which can influence mood and mitigate depressive symptoms [120]. Rostami et al. [121] conducted a study to evaluate the effects of a daily oral supplementation of 300 mg of magnesium citrate on various clinical and biochemical parameters in a cohort of 30 patients diagnosed with COVID-19. The findings of the study demonstrated a statistically significant reduction in the number of patients requiring oxygen therapy within the magnesium supplementation group (*p* < 0.001), alongside a notable enhancement in the oxygen saturation levels (*p* < 0.001). Furthermore, the participants receiving magnesium exhibited substantial improvements in their quality of life and reductions in their depression scores, evidenced by enhanced mental (MCS: *p* < 0.001) and physical component summary scores (PCS: *p* = 0.01) on the 36-item Short Form Survey (SF-36), along with a notable decrease in the Beck Depression Inventory scores (BDI: *p* = 0.03).

### 6.3. Selenium

Se is a vital trace element recognized for its antioxidant, anti-inflammatory, and immune-modulating properties. It plays a crucial role in the activity of various enzymes, functioning as a redox center and exhibiting pharmacological effects such as antiviral and immunity enhancement, dependent on concentration and compound type. This micronutrient is integral to several Se-dependent enzymes, including GPx-1 and -2 (regulating oxidative stress), GPx-4 (reducing lipid hydroperoxides), iodothyronine deiodinases (boosting immunity), and various Sels that are key to redox homeostasis and Ca signaling. Notable Sels include thioredoxin reductase; SelH and SelT (redox regulation); SelI (phospholipid biosynthesis); SelK and SelR (lymphocyte activity); SelP (cellular Se status and regulating GPx expression); SelS (protein synthesis); and selenophosphate synthetase 2 (Sel biosynthesis). Additionally, thioredoxin reductase (TrxR) plays a role in maintaining the redox status and regulating cell proliferation and immune responses [122]. Se modulates inflammatory signaling pathways by inhibiting NF-κB activity and upregulating NRF-2 signaling, which decreases inflammatory cytokine synthesis and may mitigate various diseases. Conversely, reduced serum Se levels can elevate CRP and IL-6 synthesis. Se plays a vital role in combating viral infections, as its deficiency reduces GPx activity, potentially increasing the pathogenicity of viral strains and the severity of infections, resulting in higher mortality rates compared to individuals with adequate Se levels. SelK exhibits protective effects against West Nile virus, and Trxr 1 negatively regulates HIV-1 transcriptional activator Tat in human macrophages by targeting critical disulfide bonds for transactivation. Otherwise, several viruses encode Sels, which may influence viral pathogenesis by depleting Se in host cells and compromising defenses against lipid peroxidation and membrane damage. In addition, the viral Sel GPx may protect against immune-induced oxidative damage, promoting immune evasion and potentially impairing viral replication. Se, through its Sels and Se-containing organic compounds, exhibits anti-COVID-19 activity by reducing ACE2 receptor expression, thus preventing SARS-CoV-2 entry, lowering pro-inflammatory cytokine release and inhibiting oxidative stress and acute respiratory distress syndrome. It also targets SARS-CoV-2 3CLPro and PLpro, which are critical for viral replication. Additionally, Se may interact with the sulfhydryl group of viral proteins, converting active sites into inactive disulfide groups, further hindering viral entry. Its anti-thrombotic and antiplatelet properties contribute to improving COVID-19 symptoms and outcomes [123]. Hafizi et al. [124] conducted a study employing nanotechnology to investigate the effects of incorporating a combination of BCc1, characterized by its iron-chelating properties (1500 mg, administered twice daily), and Hep-S Se-based nanomedicines (1500 μg, administered once daily) into the standard treatment regimen for hospitalized patients with moderate COVID-19. Over a 28-day treatment period, the results revealed a significant reduction in the IL-6 levels by 77% in the group receiving nanomedicine, in stark contrast to an 18% increase observed in the placebo group (*p* < 0.05).

## 7. Gaseous Molecules

### 7.1. Nitric Oxide

NO is a biologically active gas produced from arginine, primarily by endothelial cells. It plays a critical role in vascular homeostasis by promoting smooth muscle relaxation. NO’s uniqueness as a signaling molecule stems from its gaseous state, chemical instability, and reactivity. It exerts its effects through the intracellular cyclic 3′,5′-guanosine monophosphate (cGMP) pathway or its reactive free radical properties [125]. NO is one of the most important endogenous vascular tone regulator by activating soluble guanylate cyclase (sGC), increasing cGMP levels, reducing intracellular Ca, and inhibiting myosin light-chain kinase (MLCK), which promotes smooth muscle relaxation and improves blood flow. NO exhibits antioxidant effects by scavenging oxygen radicals like anion superoxide (O_2_^−^), inhibiting H_2_O_2_ production, and enhancing intracellular GSH levels through the activation of γ-glutamylcysteine synthetase, the rate-limiting enzyme in GSH synthesis. Additionally, NO inhibits the redox-sensitive transcription factors NF-κB and IRF-1, reducing the expression of pro-inflammatory genes. Recent evidence highlights NO’s antiviral activity, particularly against SARS-CoV, by inhibiting viral replication and RNA synthesis [126]. Its pulmonary vasodilation improves oxygenation, creating an inhospitable environment for the virus. Inhaled NO (iNO) has been shown to reverse pulmonary hypertension, alleviate severe hypoxia, and shorten ventilatory support duration in SARS-CoV patients [127]. Approved by the FDA in 1999 for neonatal hypoxic respiratory failure and severe ARDS, iNO has demonstrated benefits such as bronchodilation, inflammation suppression, and antimicrobial effects, potentially reducing hospital stays in viral respiratory infections [128]. The iNOS-mediated production of NO is essential for the inflammatory response, particularly during viral infections, in which it is upregulated by IFN-1. This enhancement is facilitated through TLRs that recognize viral PAMPs like double-stranded viral RNAs (dsRNAs), promoting iNOS expression via the NF-κB pathway. NO exerts an inhibitory effect on SARS-CoV host cell infection in a concentration-dependent manner, primarily by reducing the palmitoylation of the S protein and obstructing the ACE2-mediated fusion process. Additionally, NO influences viral RNA replication through the suppression of cysteine protease activity, specifically targeting the S-nitrosation of the 3CL protease encoded by ORF1a. Additionally, NO generates reactive RNS such as peroxynitrite (ONOO^−^), disrupting both viral DNA and RNA replication by modifying cysteine residues, impairing transcription, and causing oxidative damage to both viral and host genomes, particularly in viruses lacking repair mechanisms [129]. A review of 14 studies involving 423 COVID-19 patients found iNO modestly improved oxygenation (as indicated by increased PaO2/FiO2 ratios) in some cases but did not significantly impact mortality [130]. Fakhr et al. [131] demonstrated the safety and efficacy of high-dose iNO (160 ppm twice daily) in non-intubated COVID-19 pneumonia patients, using a specialized mask. This approach improved oxygenation and reduced the need for hospital readmission, suggesting broader benefits of NO beyond enhancing pulmonary blood flow.

### 7.2. Ozone

O_3_ is a triatomic molecule with dynamic instability due to mesomeric states, making it a potent oxidizing agent. This reactivity is beneficial in therapeutic applications, such as ozone therapy, which has been used since World War I to treat infections and promote wound healing [132]. O_3_ therapy has been explored for its potential effects on inflammation and immune regulation in COVID-19 [133]. It modulates the NLRP3 inflammasome, which drives inflammation in severe infections, helping reduce excessive inflammation [134]. O_3_ also interacts with plasma antioxidants, generating H_2_O_2_ that boosts immune responses. In vitro studies suggest O_3_ disrupts lipid-enveloped viruses by oxidizing lipoproteins and glycoproteins, hindering viral entry [135]. Additionally, ozone reduces pro-inflammatory cytokines like IL-1, IL-6, and TNF-α, counteracting the hyperinflammation seen in severe COVID-19 [134]. O_3_ may also block ACE2 receptors used by SARS-CoV-2 to enter cells, potentially via NRF-2 pathway activation [136]. Its oxidative effects have the potential to inhibit the viral fusion process by modifying the cysteine-rich regions at the C-terminal of the S2 domain, thereby reducing the virus’s ability to infect host cells [137]. In a study by Hernández et al. [138], ozonated autohemotherapy combined with standard care led to significantly better outcomes in nine COVID-19 patients, including faster clinical improvement and a quicker negative CRP test. The ozonated group also showed a more rapid reduction in biomarkers like CRP, ferritin, D-dimer, and LDH.

## 8. Conclusions

The complex interaction between SARS-CoV-2 and cellular redox mechanisms underscores the potential of antioxidants in mitigating COVID-19’s effects. SARS-CoV-2 manipulates the redox machinery to promote viral replication, trigger inflammation, and induce apoptosis, leading to tissue damage and organ complications. Key pathways involving ACE2, NRF-2, and NF-κB regulate viral entry and inflammatory responses, resulting in the downregulation of antioxidant defenses. Given that increased oxidative stress is linked to severe outcomes, antioxidants may offer therapeutic benefits. Early studies suggest combining antioxidants with antiviral and anti-inflammatory treatments could improve patient outcomes. Antioxidant supplementation may indeed improve respiratory function, reduce inflammation, and shorten hospital stays, though further clinical trials are needed to optimize dosing strategies. Compounds like vitamins, trace elements, polyphenols and different cellular mediators can scavenge ROS, supporting endothelial function, reducing thrombosis, and mitigating cytokine storms, all of which contribute to COVID-19 morbidity and mortality. In addition to their various effects, certain compounds possess direct antiviral capabilities by inhibiting the viral fusion process and host cell entry. This is achieved by binding to key viral protein targets, such as ACE2, furin, or TMPRSS2. Furthermore, these compounds can disrupt the replication process and the formation of new virions by interfering with transcription mechanisms and inhibiting the activity of critical viral proteases, including PLpro and 3CLpro, as well as the RdRp complex.

In conclusion, the pleiotropic effects of antioxidants highlight their potential in a multifaceted treatment approach. In addition to reducing oxidative stress, they may enhance immune function, aiding in SARS-CoV-2 clearance, and show synergy with antiviral therapies, enhancing efficacy with minimal adverse effects. However, rigorous randomized controlled trials are essential to determine their optimal use, and personalized antioxidant therapy tailored to individual oxidative profiles could improve outcomes worldwide, offering accessible, low-cost treatments for COVID-19 and its long-term sequelae.

A synopsis of the studies that have used some of the antioxidants discussed in hospitalized COVID-19 patients is provided in Table 1.

Table 2 summarizes the direct antiviral effects, as well as the indirect anti-inflammatory and antioxidant properties of the molecules examined in the context of COVID-19.

## Figures and Tables

**Table 1:** Antioxidant mediators tested in hospitalized patients with mild-to-moderate COVID-19 pneumonia.

Reference Study	Patients/Controls	Mediator	Administration Route	Dosage	Outcomes
Ahmadi et al., 2023 [94]	29/39	Nanocurcumin	Oral	40 mg/4 times a day for 2 weeks	↓ coughs (*p* = 0.036), ↓ fatigue (*p* = 0.0001), and ↓ myalgia (*p* = 0.027) intensity, ↓ oxygen demand (*p* = 0.036), ↓ hours of oxygen usage (*p* = 0.05), ↓ RR (*p* < 0.0001), and ↑ SpO_2_ (*p* = 0.006)
Dewan et al., 2022 [32]	118/122	GSH	Endovenous	2400 mg/daily as a loading dose followed by 1200 mg/2 times daily for 7 days	↓ symptoms (*p* < 0.001)
Erfani et al., 2023 [61]	17/17	Vitamin B_12_	Oral	1000 mg/daily for 7 days	no effect
Fakhr et al., 2021 [27]	29/0	NO	Aerosol	160 ppb/2 times daily for 30 min	↑ SpO2 (*p* < 0.05) and ↓ RR (*p* < 0.05)
Farnoosh et al., 2021 [69]	24/20	Melatonin	Oral	3 mg/3 times daily for 2 weeks	↓ symptoms (*p* < 0.05), ↓ CRP serum levels (*p* < 0.05), ↓ HRCT lung involvement (*p* < 0.05), and ↓ hospital length (*p* < 0.05)
Hafizi et al., 2023 [124]	62/60	NanoSe	Oral	1500 μg/daily of Hep-S with 1500 mg/daily of BCc1 for 4 weeks	↓ IL-6 (*p* < 0.05)
Hakamifard et al., 2021 [89]	38/34	Vitamin E	Oral	400 UI/daily of vitamin E with 1g/daily of vitamin C for 7 days	no effect
Hernández et al., 2020 [62]	9/9	O_3_	Endovenous	200 mL autologous whole blood enriched with 200 mL of O_2_-O_3_ mixture with a 40 μg/mL O_3_ concentration/2 times daily for 4 days	↑ clinical improvement (*p* = 0.04), ↓ time to negative PCR for SARS-CoV-2 testing (*p* = 0.04), ↓ CRP (*p* = 0.008), ↓ ferritin (*p* = 0.016), ↓ D-dimer (*p* = 0.009), and ↓ LDH (*p* = 0.01) serum levels
Jahangirifard et al., 2021 [38]	20/20	Bromelain	Oral	200 mg/3 times daily for 5 days	↑ SpO2 (*p* < 0.05), ↑ RR (*p* < 0.05), ↑ HR (*p* < 0.05), ↑ AST (*p* < 0.05), ↑ ALT (*p* < 0.05), ↑ BUN (*p* < 0.05), ↑ ESR (*p* < 0.05), ↑ LDH (*p* < 0.05), ↑ WBC (*p* < 0.05), and ↑ lymphocyte count (*p* < 0.05)
Rostami et al., 2024 [121]	30/30	Mg citrate	Oral	300 mg/daily	↓ O_2_ therapy (*p* < 0.001), ↑ SpO_2_ (*p* < 0.001), ↑ SF-36 MCS (*p* < 0.001), ↑ SF-36 PCS (*p* = 0.01), and ↓ BDI-II (*p* = 0.03)
Sabico et al., 2021 [38]	36/33	Vitamin D	Oral	150 µg/daily vs. 25 µg/daily for 2 weeks	↓ cough (*p* = 0.039), ↓ ageusia (*p* = 0.035) duration, and ↓ hospital length (*p* = 0.039)
Sanduzzi et al., 2022 [96]	43/0	Resveratrol	Aerosol	4.75 mg/3 times daily for 2 weeks	↑ P/F ratio (*p* = 0.033), ↑ SpO_2_ (*p* < 0.001), ↓ ICU admission (*p* < 0,05), ↓ CPR (*p* < 0.001), ↓ IL-6 (*p* < 0.001), and ↓ fibrinogen (*p* < 0.001) serum levels
Shohan et al., 2021 [85]	30/30	Quercetin	Oral	500 mg/2 times daily for 7 days	↓ hospital length (*p* = 0.039), ↓ ALP (*p* = 0.002), ↓ CRP (*p* = 0.004), and ↓ LDH (*p* = 0.032) serum levels
Somi et al., 2022 [58]	15/15	Vitamin A	Intramuscular	50,000 IU/day for 2 weeks	no effect
Taher et al., 2021 [55]	47/45	NAC	Intravenous	40 mg/kg/day for 3 consecutive days	no effect
Talebi et al., 2022 [35]	32/43	L-carnitine	Oral	1000 mg/3 times daily for 5 days	↑ SpO_2_ (*p* = 0.039), ↓ ESR (*p* = 0.021), ↓ CRP (*p* = 0.009), ↓ Hb (*p* = 0.026), ↓ ALP (*p* = 0.010), ↓ LDH (*p* = 0.002), ↓ CPK (*p* = 0.019), and ↓ mortality (*p* = 0.030)
Tehrani et al., 2022 [48]	18/26	Vitamin C	Intravenous	2 g/4 times daily for 5 days	↑ SpO_2_ (*p* = 0.02), ↓ RR (*p* = 0.03), and ↓ HRCT lung involvement (*p* = 0.02)
Yao et al., 2020 [117]	40/14	Zn sulfate	Oral	440 mg/daily	no effect

Abbreviations: RR, respiratory rate; SpO2, oxygen saturation; NO, nitric oxide; CRP, C-reactive protein; HRCT, high-resolution computed tomography; Se, selenium; IL-6, interleukin-6; O_3_, ozone; O_2_, oxygen; PCR, polymerase chain reaction; SARS-CoV-2, Severe Acute Respiratory Syndrome Coronavirus 2; LDH, lactate dehydrogenase; P/F ratio, PaO2/FiO2; HR, heart rate; AST, aspartate aminotransferase; ALT, alanine aminotransferase; BUN, blood urea nitrogen; ERS, erythrocyte sedimentation rate; WBC, white blood cells; Mg, magnesium; SF-36 MCS, 36-Item Short Form Survey Mental Component Summary; SF-36 PCS, 36-Item Short Form Survey Physical Component Summary; BDI-II, Beck Depression inventory-II; ICU, intensive care unit; ALP, alkaline phosphatase; NAC, N-acetylcysteine; Hb, mean hemoglobin; CPK, creatine phosphokinase; Zn, zinc.

**Table 2:** Analysis of antioxidant molecules and their antiviral, anti-inflammatory, and immune-enhancing properties in COVID-19.

Antioxidant Molecule	Anti-Inflammatory or Immune-Enhancing Effects	Direct SARS-CoV-2 Inhibitory Effects
Bromelain	↑ macrophages, NK cells, and lymphocyte activity through the activation of PI3K/Akt and MAPK pathways ↓ cytokine storm (IL-6, TNF-α, bradykinin, and PGE2) by limiting iNOS and COX-2 through the activation of PI3K/Akt and the suppression Raf-1/ERK-2 pathways ↑ vascular remodeling through the increase in VEGF and MMPs and the degradation of AGEr ↓ thrombosis by the inhibition of platelet aggregation and the increase in plasmin	↓ viral entry by reducing the binding between ACE2, TMPRSS2, and S protein
Curcumin	↑ Treg and ↓ Th17 cell expression, by increasing IL-10, IL-35, and TGF-β production through the TBX21 and FOXP3 pathways ↓ cytokine storm (IL-1, IL-6, IL-17, and TNF-α) by inhibiting COX-2 and iNOS through the inhibition of NF-κB and NRF-2 pathways ↓ tissue damage by downregulating MMPs, MCP-1, MIPI-1, SDF-1, and CXCL, by inhibiting MAPKs, JNK, and NF-kB ↓ ROS (O_2_^−^, OH^−^, and H_2_O_2_) by the activation of NQO1 enzyme	↓ viral entry by reducing the binding between ACE2, TMPRSS2, and S protein ↓ viral replication through the disruption of the virion structure (inhibits the dimerization of the SARS-CoV-2 nucleocapsid C-terminal domain) and the downregulation of PLpro and RdRp activity
GSH	↑ T cell activation (shifts the cytokine response from Th2 to Th1), neutrophil phagocytosis, and dendritic cell functions of antigen presentation (enhances macrophage polarization) by promoting IL-2 and IFN-γ production ↓ ROS and RNS through the formation of GSSG	↓ viral entry by modulating the ACE2 pathway (balancing the ratio between ACE/ACE2) by the inhibition of the NF-kB
L-carnitine	↓ cytokine storm (TNF-α, IL-6, and LTB4) through the downregulation of NF-κB and NOX-1 and -2 expression and the inhibition of lipoxygenase activity ↑ T memory cell resilience by enhancing CPT1 expression ↓ ROS by decreasing MDA and by increasing GSH and SOD levels ↑ neutrophil and macrophage functions through the modulation of G6PD and MIF1 production ↑ CD4+ and CD8+ T cells by reducing lymphocyte apoptosis through the downregulation of Fas signaling and ceramide production	↓ viral entry by reducing the expression of ACE2, TMPRSS2, and furin by upregulating HNF4-α
Mg	↑ NK cells and CD8+ T lymphocyte cytotoxic activities through the modulation of the NF-κB pathway ↑ mastocyte, neutrophil, and macrophage activity by the inhibition of the TLR pathway ↓ ROS by decreasing MDA and by increasing SOD levels	↓ viral entry by reducing the binding of TMPRSS2 and by modulating ACE2 pathway (balancing the ratio between ACE/ACE2) via PAF inhibition
Melatonin	↑ NK and CD4+ and ↓ CD8+ cells through the NF-κB pathway ↑ vascular integrity by the inhibition of adhesion and migration of immune cells by downregulating LTB4 and IL-1β ↓ ROS and RNS by increasing SOD and GSH ↓ cytokine storm (IL-1β, IL-6, IL-8, IL-18, and TNF-α) by inhibiting the NLRP3 inflammasome, iNOS, COX-2, and TLR4 activation by NF-κB signaling	↓ viral entry by reducing the binding of ACE2 and blocking calmodulin ↓ viral replication through the downregulation of 3CLpro activity and the formation of hyperinflammatory macrophages by SIRT-1 activation
O_3_	↓ cytokine storm (IL-1, IL-6, and TNF-α) through the modulation of NLRP3 ↓ ROS by increasing SOD, CAT, and HO-1 expression via NRF-2 pathway ↑ immune responses by H_2_O_2_ generation	↓ viral entry by reducing the binding of ACE2 via NRF-2 pathway activation, altering virus’s S2 protein fusion process, and oxidizing envelope lipoproteins and glycoproteins ↑ virus immune recognition by altering glycosidic pericapsid
NAC	↓ cytokine storm (TNF-α, TGF-β, IL-1, and IL-8) and adhesion molecules (VCAM-1 and E-selectin) through NF-kB and MAPK inhibition ↑ CD8 + T lymphocyte cytotoxic activities through the modulation of the NF-κB pathway ↓ ROS (OH^−^ and H_2_O_2_) by increasing SOD and GSH synthesis ↑ vascular integrity by increasing NO bioavailability	↓ viral entry by reducing the binding between ACE2 and S protein through the action of its thiol groups ↓ viral replication through the downregulation of 3CLpro and PLpro activity and enhancing IFN-1 and H_2_S production via TLR7 and MAVS
NO	↓ cytokine storm (IL-2 and INF-1) and Th1 cells by inhibiting NF-κB and IRF-1 pathways ↓ ROS by increasing GSH through γ-GCS activation	↓ viral entry by reducing the binding between ACE2 and S protein ↓ viral replication through the downregulation of 3CLpro activity and through the disruption of viral genome by RNS generation
Quercetin	↓ cytokine storm (TNF-α, INF-γ, IL-1β, and IL-6), suppressing NLRP3 inflammasome activation and NF-κB pathway ↓ adhesion molecules (ICAM-1), chemokines (CXCL-1 and CXCL-2), and pro-inflammatory mediator (MCP1, IP-10, RANTES, GM-CSF, G-CSF, VEGF, PGE2, COX-2, and iNOS) expression through MAPK pathway ↓ ROS, RNS, and lipoperoxidation by inhibiting XO and NOX activity via NRF-2 pathway ↓ thrombosis by the inhibition of PDI	↓ viral entry by reducing the binding between ACE2, furin, TMPRSS2, and S protein and modulating the acid sphingomyelinase/ceramide system ↓ viral replication through the downregulation of 3CLpro, PLpro, RdRp, and RHA activity
Resveratrol	↓ cytokine storm (TNF-α, IL-1β, IL-6, and IL-17) suppressing NF-κB and MAPK pathway ↑ macrophages, NK, and CD4+ and CD25+ T cell activity ↓ virus-induced apoptosis by the downregulation of FGF-2 ↓ ROS and RNS by activating eNOS, NQO1, SOD, and GSH S-transferase and by inhibiting COX-2, NOX, and PPAR-γ via NRF-2 activation ↓ thrombosis by the inhibition of SIRT-1 pathway	↓ viral entry by reducing the binding of ACE2 ↓ viral replication through the downregulation of RdRp, PLpro, and 3CLPro activity and reducing the N protein expression
Se	↓ cytokine storm (CRP, TNF-α, IL-1, and IL-6) by inhibiting NF-κB activity and upregulating NRF-2 signaling ↑ NK, T, and B cell activity by upregulation of IL-2 receptor ↓ ROS and RNS by modulating GPx and TrxR activity ↓ thrombosis, inhibiting NF-κB	↓ viral entry by reducing the binding of ACE2 through the interference of viral proteins’ sulfhydryl groups ↓ viral replication, inactivating 3CLPro and PLpro
Vitamin A	↓ cytokine storm (TNF-α, NO, PGE2, COX-2, and IL-12) via RAR, RXR, and PPAR-β ↑ Treg and B cell differentiation while ↓ Th-17 cells modulating EGFR, IL-5, IL-10, IL-22, IFN-γ, and ICAM-1 via MAPK-1 and PRKCB pathway ↓ ROS and RNS by increasing CAT, SOD, and GSH through NRF-2 pathway	↓ viral replication, increasing dsRNA recognition by cytosolic receptor (RIG-I and MDA5) and IFN-α production via the NF-kB pathway
Vitamin B_12_	↓ cytokine storm (TNF-α, IL-1, IL-6, IL-17A, CCL-1, CCL-3, and CXCL-9), inhibiting NF-κB ↑ CD8+ T and NK cells through NF-κB pathway ↓ ROS by increasing GSH and GSSG via modulating coenzyme-B_12_	↓ viral entry by reducing the binding between ACE2, furin, DPP-4, and hAPN ↓ viral replication, inactivating 3CLPro and RdRp
Vitamin C	↓ cytokine storm (TNF-α, IL-1, IL-4, and IL-6) by downregulating NF-kB ↑ T CD4 +, T CD8+, B, and NK cell activity, phagocyte migration, and IFN-α, INF-β, and IFN-γ production (shifts the cytokine response from Th2 to Th1 and induces Th17 polarization) through the JAK1/STAT1 pathway ↓ ROS and RNS by increasing GSH, CAT, and NADPH through NF-kB pathway ↓ thrombosis by blocking pathways involved in the formation of NETs	↓ viral entry by reducing ACE2, furin, and cathepsin L expression ↓ viral replication inactivating 3CLPro and RdRp
Vitamin D	↓ cytokine storm (TNF-α, IL-1, IL-6, and IL-8) by downregulating NF-kB and influencing the NLRP3 inflammasome ↑ T CD4 + and T CD8+ cell activity (shifts the cytokine response from Th1 and Th17 to Th2 and Treg response) ↑ antimicrobial and antiviral protein (β-defensin 2 and cathelicidin) production ↓ ROS by suppressing NOX and increasing SOD and CAT expression ↑ vasodilatation through RAS suppression	↓ viral entry, modulating the ACE2 pathway (balancing the ratio between ACE/ACE2) by the inhibition of the NF-kB
Vitamin E	↓ cytokine storm (TNF-α, IL-1, IL-6, NO, and PGE2) by COX-2 inhibition ↑ CD4+ T, B, and NK cell activity (modulation of the Th1/Th2 balance) ↓ monocyte, macrophage, and neutrophil proliferation through PKC-α ↓ ROS and RNS by preventing lipid peroxidation and maintaining membrane integrity	↓ viral replication, inactivating 3CLPro and RdRp activity
Zn	↓ cytokine storm (TNF-α, CRP, IL-1β, and IL-6) by inhibiting NF-kB pathway ↑ macrophage and Th1 and NK cell function by increasing cytokine synthesis (IFN, IL-2, and IL-12), increasing the Bcl-2/Bax ratio ↓ ROS and RNS by suppressing NOX	↓ viral entry reducing ACE2 expression and activity ↓ viral replication, preventing RNA synthesis and modulating cytokine mRNA by the downregulation of RdRp activity

Abbreviations: NK, natural killer; PI3K/Akt, phosphoinositide 3-kinase/protein kinase B; MAPK, mitogen-activated protein kinase; IL-6, interleukin 6; TNF-α, tumor necrosis factor-α, PGE2, prostaglandin E2; iNOS, inducible nitric oxide synthase; COX-2, cyclooxygenase 2; Raf-1/ERK-2, proto-oncogene serine/threonine-protein kinase/extracellular-regulated-kinase-2; VEGF, vascular endothelial growth factor; MMPs, matrix metalloproteinases; AGEr, advanced glycation end-product receptor; ACE2; angiotensin-converting enzyme 2; TMPRSS2, transmembrane serine protease 2; S, spike; Treg, regulatory T cell; Th, T helper; TGF-β, transforming growth factor-β; TBX21, T-box transcription factor 21; FOXP3, forehead box protein 3 gene; NF-κB, nuclear factor-kappa B; NRF-2, nuclear factor erythroid-related factor 2; MCP-1, monocyte chemoattractant protein-1; MIPI-1, macrophage inflammatory protein-1; SDF-1, stromal cell-derived factor-1; CXCL, CXC motif chemokine; JNK, Jun N-terminal kinase; ROS, reactive oxygen species; O_2_^−^, anion superoxide; OH^−^, hydroxyl radicals; H_2_O_2_, hydrogen peroxide; NQO1, NADPH quinone oxidoreductase 1; PLpro, papain-like protease; RdRp, RNA-dependent RNA polymerase; GGH, glutathione; IFN-**γ**, interferon-**γ**; GSSG, oxidized glutathione; LTB4, leukotriene B4; NOX, NADPH oxidase; CPT-1, carnitine palmitoyltransferase; MDA, malondialdehyde; SOD, superoxide dismutase, G6PD, glucose 6-phosphate dehydrogenase; MIF-1, macrophage inhibitory factor-1; HNF4-α, hepatocyte nuclear factor 4-α; Mg, magnesium; TLR, Toll-like receptor; PAF, platelet-aggregating factor; RNS, reactive nitrogen species; NLRP3, NOD-like receptors family pyrin group containing 3; SIRT-1, sirtuin-1; O_3_, ozone; CAT, catalase; HO-1, hemeoxygenase; NAC, n-acetylcysteine; VCAM-1, vascular cell adhesion molecule-1; NO, nitric oxide; INF-1, infermeron-1; H_2_S, hydrogen sulfide; MAVS, mitochondrial antiviral signaling; γ-GCS, γ-glutamylcysteine synthetase; IRF-1, and interferon regulatory factor 1; Th1, helper T cells; 3CLpro, 3-chymotrypsin-like protease; ICAM-1, intercellular adhesion molecule-1; IP-10, interferon-γ-induced protein-10; RANTES, regulated upon activation normal T cell expressed and secreted; GM-CSF, granulocyte macrophage colony-stimulating factor; G-CSF, granulocyte colony-stimulating factor; XO, xanthine oxidase; PDI, protein disulfide isomerase; RHA, RNA helicase; FGF-2, fibroblastic growth factor; eNOS, endothelial nitric oxide synthase; PPAR-γ, peroxisome proliferator-activated receptor-γ; N, nucleocapsid; Se, selenium; CRP, C-reactive protein; GPx, GSH peroxidase; TrxR, thioredoxin reductase; RAR, retinoic acid receptor; RXR, retinoid receptor X; EGFR, epidermal growth factor receptor; PRKCB, protein kinase C-β; dsRNA, double-stranded RNA; RIG-I, retinoic acid-inducible gene I; MDA5, melanoma differentiation-associated protein 5; DPP-4, dipeptidyl peptidase-4; hAPN, human aminopeptidase N; NAPDH, nicotinamide adenine dinucleotide phosphate; JAK/STAT, janus kinase/signal transducers and activators of transcription; NET, neutrophil extracellular trap; RAS, renin-angiotensin system; PKC-α, protein kinase C-α; Bcl-2, B-cell leukemia/lymphoma 2 protein; Bax, Bcl-2 associated X; Zn, Zinc.

## Data Availability

No new data were created or analyzed in this study. Data sharing is not applicable to this article.
